# Factors Influencing Residents' Psychological Status During Standardized Training in COVID-19

**DOI:** 10.3389/fpsyt.2021.737717

**Published:** 2021-11-17

**Authors:** Dongfang Xiang, Bao Liang, Yongyi Wang, Biao Li, Juan Peng, Sheng Zhang, Bei Chen, Chuandong Yan, Chao Xu

**Affiliations:** ^1^Department of Psychiatry, Wuhan Mental Health Center, Wuhan, China; ^2^Affiliated Wuhan Mental Health Center, Tongji Medical College, Huazhong University of Science and Technology, Wuhan, China; ^3^Wuhan Hospital for Psychotherapy, Tongji Medical College, Huazhong University of Science and Technology, Wuhan, China; ^4^Tongji Medical College, Huazhong University of Science and Technology, Wuhan, China

**Keywords:** residents, psychological status, standardized training mode, family economic, satisfaction

## Abstract

**Objectives:** To explore the influencing factors of residents' psychological status during standardized training in COVID-19 for finding ways to promote their mental health.

**Methods:** A total of 760 residents were surveyed with a structured questionnaire. Correlation analysis was used to analyze the influencing factors of psychological status of the residents, and a mediation model was constructed to verify the mediating role of satisfaction.

**Results:** Age, willingness to study medicine, and satisfaction were positively correlated with negative psychological status (*P* < 0.05). And gender, only child or not, and annual household income (RMB) were negatively correlated with negative psychological status (*P* < 0.01). Residents' satisfaction with standardized training mode plays a complete mediating role between annual household income and negative psychological status.

**Conclusions:** Our findings emphasize the importance of concentrating on resident's psychological status and family economic situation. And relative departments should take action to optimize the standardized training mode to improve the satisfaction.

## Introduction

The world has been facing a pandemic of Corona Virus Disease 2019 (COVID-19), and this public health emergency was first reported in Wuhan, China, at the end of 2019 (1). At present, some countries have entered a stable period, but some countries are still in the epidemic period. Plenty of experts and scholars pointed out that this disease not only affects physical health, but also seriously affects mental health (2), such as depression, anxiety, mood disorders, sleep disorders, post-traumatic stress disorder, and so on (3). Some scholars paid special attention to the medical staff (4), standardized training residents of whom are a special group. They are in the process of standardized training while preventing and controlling infection. However, due to the competitive pressure, insufficient economic ability, heavy learning and family burden, their psychological pressure will be much higher than that of the general medical staff. A previous study evaluated the impact of the COVID-19 pandemic on the training program for obstetrics and gynecology residents in Italy, and the results showed that 84% of residents reported anxiety about their career future (5).

Standardized training in China started late and the system is not yet comprehensive, causing many residents to be dissatisfied with the training model, then lead to psychological pressure. According to a survey in 2015, only 33% of trainees were satisfied with the standardized training mode in Shenzhen, China (6). Similarly, 183 students do not support the standardized training system among the 600 undergraduate students who majored in clinical medicine. The main reason is that the income during the standardized training cannot meet their expenses (7). Smith et al. reported that Oral and Maxillofacial Surgery Residents' increased stress was related to significantly decreased odds of satisfaction (8). And job satisfaction has the strongest association with mental/psychological problems, such as burnout and depression (9).

Family factors are also often reported to be associated with negative attitudes, with family economic situation being most often concerned. Family income instability was shown to worsen depression among college students during COVID-19 epidemic (10), which is similar to Fadilah et al.'s findings (11).

Standardized training is an important part of post-graduation education for medical students and is extremely important for training high-level clinical physicians and improving the quality of medical care. Residents can experience the whole standardized training process most directly, and their satisfaction with the training model and mental health are very worth exploring and improving, but there are few researches on the mechanism. This study focuses on the psychological status of residents during standardized training in the public health emergency, and aims to analyze the impact of residents' family economic situation and their satisfaction with the training model on their psychological status, for providing the guidance for promoting mental health.

## Methods

### Participants

This study was a cross-sectional study using non-probability convenience sampling to select samples among residents during standardized training in four tertiary hospitals in Wuhan, Hubei Province (Tongji Hospital, Union Hospital, Zhongnan Hospital and Renmin Hospital) from January 2021 to April 2021. The sample size was estimated using the calculation formula of cross-sectional survey: N ≥ ${\text{Z}}_{1-\text{α}/2}^{2}$p(1-p)/δ^2^, α = 0.05, Z_1−α/2_ = 1.96, *p* = 35% [according to a research in 2021(12)], δ = 0.1p. Respondents completed an online self-designed anonymous questionnaire and a total of 760 residents participated in this survey, which meets the requirement for sample size. And our questionnaire response rate was good (100%). This study was approved by the Ethics Committee of Wuhan Municipal Health Committee (KY2018.26) and we had obtained informed consent from the interviewees before conducting the survey.

### Measures

Annual family income, psychological status, and satisfaction of standardized training mode (henceforth referred to as satisfaction) were assessed by different items.

Annual family income (RMB) 1 ≤ 50,000, 2 = 50,000–100,000 (including 50,000), 3 = 100,000–150,000 (including 100,000), 4 ≥ 150,000.

Psychological status was measured using the DASS-42 (test-retest reliability: 0.884, interval is 2 weeks) (13). DASS-42 was developed in 1995 by Lovibond and Lovibond (14) and Chinese version of DASS-42 was provided by the original author with good internal consistency, face, and content validity. The DASS-42 are divided into three subscales and each subscale consists of 14 questions, which are the depression subscale, the anxiety subscale, and the stress subscale. Each item was scored using a Likert four-point scale ranging from 0 = Did not apply to me at all to 3 = Applied to me very much or most of the time. Table 1 presents the scoring criteria for the degree of depression, anxiety and stress.

**Table 1:** Sample characteristics (*N* = 760).

**Categorical variables**	**Mean ± SD/n (%)**
Age (years)	26.60 ± 1.92
**Gender**	
Male	373 (49.1)
Female	387 (50.9)
**Only child or not**	
Yes	508 (66.8)
No	252 (33.2)
**Married or not**	
Married	118 (15.5)
Unmarried	642 (84.5)
**Willingness to study medicine**	
My will	596 (78.4)
Not my will	164 (21.6)
**Annual household income (RMB)**	
<50,000	289 (38.0)
50,000–100,000 (including 50,000)	297 (39.1)
100,000–150,000 (including 100,000)	112 (14.7)
>150,000 (including 150,000)	62 (8.2)
**Family member with medical background**	
Yes	157 (20.7)
None	603 (79.3)
**Satisfaction with standardized training mode**	
Very satisfied	1 (0.1)
Satisfied	43 (5.7)
Doesn't matter	61 (8.0)
Not satisfied	319 (42.0)
Very dissatisfied	336 (44.2)

Satisfaction Respondents were asked about their satisfaction with standardized training mode (1 = very satisfied and 5 = very dissatisfied).

Other variables Family factors include only child (1 = Yes, 2 = No), marriage (1 = Married, 2 = Unmarried), willingness to study medicine (1 = My will, 2 = Not my will), family members with a medical background (1 = Yes, 2 = None).

**Figure 1:**
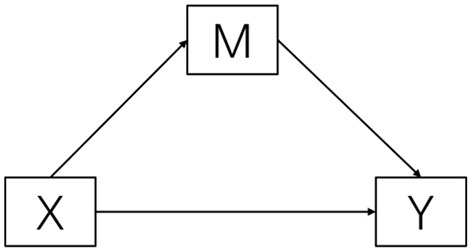
Conceptual diagram.

Statistical analysis was conducted using the SPSS 24 version program. The descriptive analysis was used to determine the demographic characteristics of the participants. Pearson correlation analyses of the study variables were conducted. The SPSS Process was used to test the effects of family income on psychological status through satisfaction. The bootstrapping method was used to verify mediation effects. In this study, we bootstrapped 5,000 samples from the data, and 95% bootstrap confidence intervals (CI) were calculated. The conceptualized model was shown in Figure 1.

## Results

### Sample Characteristics

The mean age of the participants was 26.60 years (SD = 1.92). The sample consisted of 760 residents. 49.1% (*n* = 373) were male. A total of 508 (66.8%) participants were only child. Majority of respondents followed their own wishes when choosing a medical specialty. Over 80% participants were not satisfied or very dissatisfied with the standardized training mode. Table 1 shows the detail of sample characteristics.

### Negative Psychological Status of Residents

Of all respondents, 47.0% were depressed, 49.3% were anxious, and 48.4% were stressed. And more than 10% were in very terrible condition, of which 83 were in a very severely depressed state, 105 were extremely anxious and 97 were extremely nervous. Specific scores and degrees of depression, anxiety and stress (Table 2).

**Table 2:** Scores and degrees of depression, anxiety, and stress.

	**Score**	**Degree**	***n* (%)**
Depression	11.92 ± 9.87	1	403 (53.0)
		2	93 (12.2)
		3	115 (15.1)
		4	66 (8.7)
		5	83 (10.9)
Anxiety	10.34 ± 8.876	1	385 (50.7)
		2	45 (5.9)
		3	139 (18.3)
		4	86 (11.3)
		5	105 (13.8)
Stress	16.21 ± 12.450	1	392 (51.6)
		2	67 (8.8)
		3	118 (15.5)
		4	86 (11.3)
		5	97 (12.8)
Total	38.471 ± 30.651		760 (100)

### Correlation Analysis

In our study, gender, only child or not, willingness to study medicine and satisfaction with standardized training mode were most significant influencing factors (*P* < 0.01). Age was positively correlated with negative psychological status (*P* < 0.05). And annual household income (RMB) was negatively correlated with negative psychological status (*P* < 0.01). More details were presented in Table 3.

**Table 3:** Correlation analysis.

	**Depression**	**Anxiety**	**Stress**
Age	0.085^*^	0.103^**^	0.097^**^
Gender	−0.248^***^	0.222^***^	0.235^***^
Only child or not	−0.215^***^	0.226^***^	0.221^***^
Married or not	−0.068	−0.095^**^	−0.075^**^
Willingness to study medicine	0.189^***^	0.174^***^	0.204^***^
Annual household income	−0.108^**^	−0.098^**^	−0.098^**^
Family member with medical background	0.060	0.065	0.046
Satisfaction with standardized training mode	0.239^***^	0.233^***^	0.237^***^

* *P < 0.05*,

** *P < 0.01*,

*** *P < 0.001*.

### Mediation Analysis of Satisfaction on Negative Psychological Status

Table 4 showed total effect, direct effect and mediation effect, which revealed a mediation role of satisfaction in the relationship between annual household income and negative psychological status. The direct effect of annual household income on negative psychological status was negative and not significant, with the CI from −4.333 to 0.301. However, the indirect effect through satisfaction is significant with CI from −1.306 to −0.226, indicating the role of complete intermediary of satisfaction.

**Table 4:** Total, direct, and indirect effect.

	**Effect**	**BootSE**	**BootLLCI**	**BootULCI**
Indirect effect	−0.711	0.282	−1.306	−0.226
Direct effect	−1.996	1.182	−4.333	0.301
Total effect	−2.707	1.159	−4.950	−0.400

## Discussion

### Psychological Status of Residents

The results of the study showed that the psychological status of medical students during COVID-19 was not very optimistic in general. There may be three obvious reasons for such a result. The most direct reason is that the workload has increased and the work process has become complicated during COVID-19. Compared with healthcare workers who have not been in contact with COVID-19 patients in the workplace, those who encountered COVID-19 patients faced more task load among Iranian medical staff (15). For instance, the application of personal protective equipment is very necessary for preventing infection, but the use of protective equipment greatly increases the workload and fatigue of healthcare workers (15, 16). Simultaneously, Liu et al. emphasized that intensive work can increase physical and emotional stress (17). In addition to daily medical work, residents also need to participate in regular training, while preparing various types of examinations. The exam pattern was changed because of the epidemic and medical courses or training programs are difficult and challenging, they need to contend with new test formatting in a short period, which may cause higher levels of examination anxiety (18, 19). Second, the income of residents is low during the training period (20). Low personal income may lead to dissatisfaction with life (21). And low socioeconomic status (SES), which includes per capita household income, is directly associated with increased mental health problems in children and adolescents (22, 23). Children and adolescents with low SES are two to three times more likely to have mental health problems than their peers with high SES (24). Finally, it is worth mentioning that we speculate that age may also be an influencing factor. Non-medical peers may already have independent financial capacity and work achievement. In contrast, medical students may feel additional pressure.

### Factors Influencing Psychological Status of Residents

#### Age

Numerous studies have demonstrated that younger individuals are more likely to produce negative psychological status (3). For example, Losada-Baltar et al. found that older adults reported lower levels of anxiety and sadness than middle aged adults, and middle-aged adults reported lower levels than younger participants (25). Moreover, Liu et al. indicated that larger (more negative) error-related negativity associated with more anxiety in older girls, whereas smaller error-related negativity associated with more anxiety symptoms in younger girls (26).

#### Gender

It seems that people all have a mindset that women always seem to be perceived as emotional, so their emotions are usually unstable and more likely to be negative (27). But our study showed the opposite results. Some experts believe that males may suppress the expression or release of emotions, but in fact they are experiencing psychological pain (28). So, our results may be more real.

#### Only Child or Not

Residents with siblings perform better when socialized and also cope better in crisis situations (29), and non-only children will receive more support when encountered difficulties.

#### Marriage

Studies have shown that married patients show better psychological adaptation and physical health (30). People with a spouse may be more likely to receive trustworthy emotional support in a dilemma. Specifically, research on help-seeking behavior has demonstrated that people think of their partners when they need help (31). The result is similar to Becker et al.'s study, who found that in both the children's network and the family network, the second major support comes from the partner (32).

#### Willingness to Study Medicine

Gu (33), from Shihezi University, found that when Chinese high school students choose college majors, they will finally prefer parental decisions rather than their own decisions. That is, some medical students may be reluctant or have insufficient interest when they enter the medical field at the beginning. Interest in learning is the starting point of education, as it can motivate students to learn, and students' learning performance can be significantly improved through their interest in learning. If a person's interest in learning can be improved, then the person's cognitive function, perseverance, and affect can be enhanced (34). Consequently, it may not be enough to support them to maintain a good positive attitude in subsequent learning if the medical specialty is not their own will.

#### Annual Household Income

Emotions can be directly or indirectly influenced by household income. Najman et al. found that family poverty is a risk factor for children to feel anxious and depressed. Namely, the higher the frequency of exposure to poverty, the higher the child's risk of feeling anxiety and depression (23). An empirical analysis showed that family income impact children's health by being significantly associated with parents' emotional well-being (35).

#### Family Member With Medical Background

Contact with family members with medical background enables residents to receive more professional support. When seeking help, people tend to trust people who have the same experience as themselves (36). Family members with medical background have similar knowledge structure and learning experience to residents, which is conducive to providing effective support and advice and can greatly alleviate the pressure.

#### Satisfaction With Standardized Training Mode

Satisfaction has been shown to be related to psychological status. Women who are dissatisfied with relationships (such as intimacy) have higher levels of postpartum depression symptoms (37). College students' satisfaction with academic performance in Hong Kong was negatively associated with mild to severe depression (38). What's more, job satisfaction is also related to mental health (9).

### The Mediation Role of Satisfaction

The mediation result of the study showed that satisfaction was a very important mediator between annual household income and negative psychological status. Actually, economic situation is the basis of a family, and different economic conditions may lead to different family structures and family atmosphere. Moreover, a wide variety of family factors can affect a person's personality characteristics and then probably influence a people's thoughts and behaviors (39). For example, extraversion and neuroticism significantly mediated family conflict and life satisfaction in a research conducted by Xi'an Jiaotong University (40). And another study analyzed the influence of personality on psychological health and provided evidence on considering personality traits as a relevant predictor of differences in health conditions of adults during COVID-19 epidemic (41). Therefore, annual household income can have a great impact on residents' satisfaction with standardized training mode.

There were several limitations in our study. First, our study used convenience sampling, and a more precise sampling method may need to be used in future studies. Second, given the study's cross-sectional design, it is difficult to draw definitive causal conclusions regarding the long-term effect of the current pandemic. Third, selection bias cannot be excluded, and the results may not be applicable to all countries. However, the results are in line with those reported by previous similar cross-sectional study on psychological state of residents. Indeed, the study that examined the psychological impact of COVID-19 on Italian orthopedic residents found that the pandemic had an important social impact on residents' perceptions and emotional well-being (42), manifesting in the worsening by the HADS score and the depression subscale of this score after the national lockdown.

## Conclusion

The present study verified the fully mediating effect of satisfaction. Relevant departments also need to improve the training model for improving the satisfaction of residents. And Hospitals need to care about the family economic situation of residents and pay targeted attention to their psychological status.

## Data Availability Statement

The raw data supporting the conclusions of this article will be made available by the authors, without undue reservation.

## Ethics Statement

The studies involving human participants were reviewed and approved by the Ethics Committee of Wuhan Mental Health Center, Huazhong University of Science and Technology. The patients/participants provided their written informed consent to participate in this study.

## Author Contributions

DX and BLia: writing-original draft, writing-review and editing, and analyses. YW and BC: methodology. CX and SZ: conceptualization, writing-review and editing, and supervision. BLi, JP, and CY: conceptualization and analyses. All authors contributed to the article and approved the submitted version.

## Funding

This work was supported by the Wuhan Municipal Health Commission (WX18Q35).

## Conflict of Interest

The authors declare that the research was conducted in the absence of any commercial or financial relationships that could be construed as a potential conflict of interest.

## Publisher's Note

All claims expressed in this article are solely those of the authors and do not necessarily represent those of their affiliated organizations, or those of the publisher, the editors and the reviewers. Any product that may be evaluated in this article, or claim that may be made by its manufacturer, is not guaranteed or endorsed by the publisher.
